# Synergistic Effects of Ginkgolide B and Protocatechuic Acid on the Treatment of Parkinson’s Disease

**DOI:** 10.3390/molecules25173976

**Published:** 2020-08-31

**Authors:** Tingting Wu, Xianying Fang, Jiahui Xu, Yan Jiang, Fuliang Cao, Linguo Zhao

**Affiliations:** 1 Co-Innovation Center for Sustainable Forestry in Southern China, Nanjing Forestry University, Nanjing 210037, China; njfu2316@163.com (T.W.); fxy_08@163.com (X.F.); jhui6616@icloud.com (J.X.); flcao@njfu.edu.cn (F.C.); 2 College of Chemical Engineering, Nanjing Forestry University, Nanjing 210037, China

**Keywords:** Ginkgolide B, protocatechuic acid, synergistic effect, Parkinson’s disease

## Abstract

Ginkgo biloba extract (EGB) has many pharmacological activities. In the quality standard of EGB, the main quality control indexes are total flavone (content ≥ 24%) and total lactone (content ≥ 6%). There are no specific limits for nearly 70% of “other components”. In recent years, in order to pursue the production of a high-ketone ester, some enterprises removed the unwanted components, including some organic acids. Protocatechuic acid (PCA), as an important organic acid, has been reported to have a variety of biological activities. It is necessary to explore whether it can promote the biological activities of the main functional components of EGB. In this study, PCA was selected to be combined with Ginkgolide B (GB) for the treatment of Parkinson’s disease. In vitro, rotenone (rot) was used to induce PC12 cells. The survival rate was tested by the 3-(4, 5-dimethylthiazol-2-yl)-2, 5-dimethyltetrazolium bromide (MTT) assay. Reactive oxygen species (ROS) and antioxidase were detected to analyze the effects of drugs on oxidative stress. The apoptosis was tested via Western blot. The results show that the cell viability was increased, morphology was improved, the oxidative stress level decreased, and the apoptosis was inhibited after the combination treatment of GB and PCA, and the effect was better than GB or PCA alone. In vivo, MPTP (30 mg/kg) was used to induce Parkinson’s disease (PD) in male C57BL/6 mice. The motor ability of the mice was measured by pole-climbing and the suspension. The injury of nerve cells was indicated by HE staining. Oxidative stress levels were tested via antioxidant enzyme activity. The number of dopaminergic neurons was reflected by TH staining. Results show that the combination treatment of GB and PCA could significantly restore the motor ability of PD mice, reduce the injury of nerve cells, improve the activity of the antioxidant enzyme in the brain tissue, and increase the expression of TH in the substantia nigra of midbrain. Our study shows that PCA increases the efficacy of GB (the main functional ingredient of EGB) in the treatment of Parkinson’s disease, which provides a new idea for the treatment of nervous system diseases and a new concept for the efficient utilization of active components in *Ginkgo biloba* leaves.

## 1. Introduction

Ginkgo biloba is a plant of the genus *Ginkgo* in the Ginkgoaceae. Ginkgo biloba extract (EGB) has significant biological activity and important application value. It is well-recognized that lactones and flavones are the main active components of EGB [1]. Among them, Ginkgo lactones have the functions of neuroprotection, lipid-lowering abilities, and so on [2]. Ginkgo flavones have antioxidant and anti-inflammatory effects [3]. However, the other 70% of its components (including ginkgo organic acids) are often ignored or even removed by purification. Because Ginkgo organic acids are a class of active components, the necessity of its existence in EGB is worth exploring. Studies on the main active components of EGB or organic acids have been thorough. Ginkgolide B is a diterpenoid lactone [4]. It can protect nerve cells from damage and improve cognition and memory [5,6]. Protocatechuic acid (PCA), the main compound of organic acids, has many biological activities including neuroprotective and antioxidant effects [7]. Studies have shown that PCA can inhibit the apoptosis of PC12 cells and stimulate cell proliferation to treat Parkinson’s disease [8]. However, the synergistic effect of ginkgo organic acids on the ginkgo flavones or lactones has not yet been studied. Based on the function of neuroprotection, we choose to study the synergistic effect of GB and PCA in Parkinson disease (PD).

PD is a common chronic nervous system disease and most often affects the elderly. Its main clinical symptoms are the slowing of movement, resting tremor, postural instability, and rigidity. With the rapid ageing of the population, the prevention and treatment of Parkinson’s disease are urgent. Epidemiological studies show that environmental factors, such as insecticide, play an important role in the pathogenesis of Parkinson’s disease [9]. In addition, PD is closely related to mitochondrial dysfunction, oxidative stress, apoptosis, and inflammation, and these factors interact to promote its occurrence and development [10,11]. Studies have shown that the neuroprotective effects of ginkgolide B and protocatechuic acid were related to the increase of cell activity, the decrease of mitochondrial dysfunction, the inhibition of reactive oxygen species accumulation, and the decrease of apoptosis [12,13].

Rotenone (Rot)-induced apoptosis in PC12 cells has been used as an in vitro model of PD. Rot, an insecticide, is a specific inhibitor of the mitochondrial complex I [14]. Inhibitors of complex I increase ROS formation, which produces oxidative stress leading to mitochondrial dysfunction that is considered one of the major causes of neuronal cell death [15]. The rat pheochromocytoma (PC12) cell line is derived from rat pheochromocytoma tumours and exhibits many properties that are similar to those of dopamine neurons. It is one of the most widely used neuronal cell lines for research on the mechanisms of PD [16].

In vivo, 1-methyl-4-phenyl-1, 2, 3, 6-tetrahydropyridine (MPTP)-induced C57BL/6 mice are widely used as classical mouse models of PD. MPTP is a byproduct of synthetic drugs. MPP^+^ is one of its active metabolites. It selectively inhibits mitochondrial complex I and leads to mitochondrial dysfunction, which is a cause of death for dopaminergic neurons. MPTP can cause pathological and clinical manifestations similar to those of primary PD in primates, rodents, and humans.

Finally, we investigated the synergistic effects of GB and PCA by using Rot-induced PC12 cells and MPTP-induced C57BL/6 mice as models of Parkinson’s disease in vitro and in vivo. The research results are expected to provide a theoretical foundation for the quality positioning, standard formulation, and in-depth development of EGB-related products.

## 2. Results

### 2.1. The Construction of Cell Model

**Figure 1:**
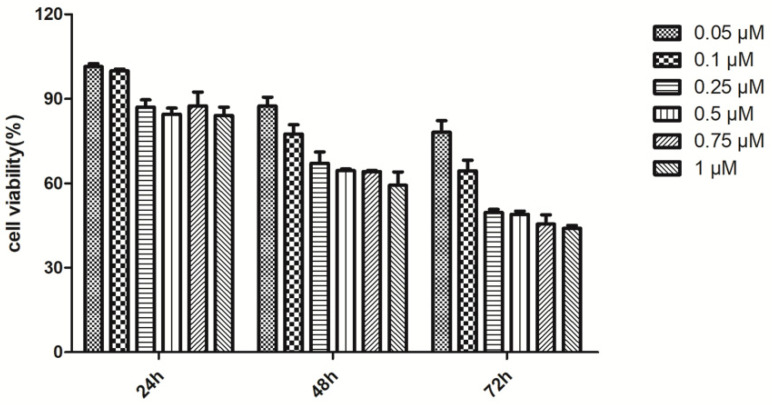
The effects of different concentrations of Rot on PC12 cell viability. PC12 cells (5 × 10^4^ cells/mL) were treated with Rot for 24, 48, and 72 h at 37 °C. Cell viability was assessed by the MTT method as described in the Materials and Methods. The data are the means ± SDs. Values were obtained for three culture wells per experiment.

As shown in Figure 1, PC12 cells were treated with Rot for 24 h, 48 h, and 72 h, respectively. Cell activity decreased as the concentration increased. When cells were treated with the 1 μM Rot for 72 h, the cell survival rate was the lowest. However, when PC12 cells were treated for 72 h, cell adhesion was greatly reduced. Thus, 1 μM rotenone treatment for 48 h was selected for the following study.

### 2.2. Effects of Drugs on Cell Viability and Cytotoxicity of Rot-Induced PC12 Cells

#### 2.2.1. The Effects of GB, PCA and Combination of Both Drugs on the Cell Viability of Rot-Induced PC12 Cells

**Figure 2:**
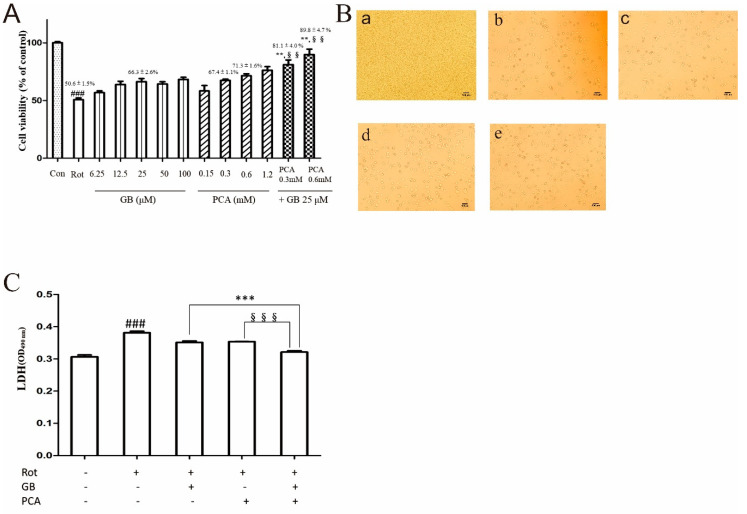
The effects of drugs on the cell viability and cytotoxicity of Rot-induced PC12 cells. (**A**) The effects of GB, PCA, and a combination of both drugs on the cell viability of Rot-induced PC12 cells. The cell viability of each treatment group was measured using an MTT assay. The data are means ± SDs. (**B**) The combined effect of GB and PCA on the morphology of Rot-induced PC12 cells (100×), a: control group; b: model group; c: GB (25 μM)-treated group; d: PCA (0.6 mM)-treated group; e: combined treatment (GB-25 μM, PCA-0.6 mM) group. (**C**) The combined effect of GB and PCA on LDH release in Rot-induced PC12 cells. The dates are expressed as the means ± SDs of the absorbance values. ^###^ *p* < 0.001 vs. the control group. ** *p* < 0.01 and *** *p* < 0.001 vs. the GB-treated group at the same concentration. ^§§^ *p* < 0.01 and ^§§§^ *p* < 0.001 vs. the PCA-treated group at the same concentration.

As shown in Figure 2A, compared with the Rot-induced PC12 cells, both the GB-treated group and the PCA-treated group reduced Rot-induced cytotoxicity in PC12 cells.

After 48 h. Notably, the cell viabilities of the combination of GB (25 μM) and PCA (0.3 mM and 0.6 mM), respectively accounting for 81.1 ± 4.0% and 89.8 ± 4.7%, were significantly enhanced compared to those of the single-treatment at the same concentration. The Q values of the combination treatment with 0.3 or 0.6 mM PCA + 25 μM GB were 1.02 and 1.18, respectively. So the combination of GB (25 μM) and PCA (0.6 mM) had a synergistic effect. This combined concentration will be adopted in the subsequent experiment.

#### 2.2.2. Combined Effects of GB and PCA on Cell Damage of Rot-Induced PC12 Cells

We investigated morphological changes in the presence or absence of the drugs to assess the protective effect of the drugs against rotenone-induced neurotoxicity. As shown in Figure 2B, under an inverted microscope, PC12 cells were well-adherent, plump, and polygonal in the normal group. After rotenone injury, the cells became round and the cell fragments increased. After drug treatment, the number of viable cells increased, and combined treatment with GB and PCA was more effective than the single treatment.

When the cytoplasmic membrane is damaged, intracellular lactate dehydrogenase (LDH) will be released into the culture medium. As shown in Figure 2C, when PC12 cells were treated with 1 μM rotenone for 48 h, the release of LDH was 0.381 ± 0.01. The combined treatment of GB and PCA reduced the release of LDH, which reached a level of 0.321 ± 0.004, which was significantly different from the single treatment groups.

### 2.3. Combined Effects of GB and PCA on Oxidative Stress in Rot-Induced PC12 Cells

**Figure 3:**
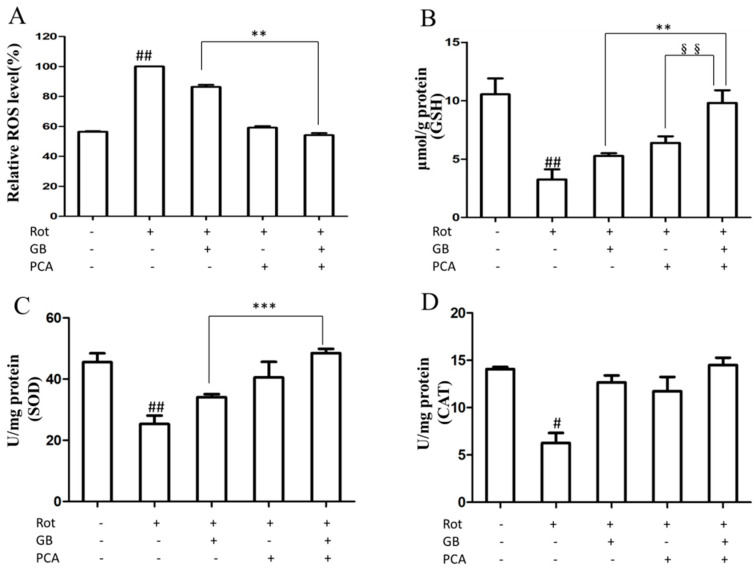
The combined effects of GB and PCA on the oxidative stress in Rot-induced PC12 cells. (**A**) The combined effects of PCA and GB on the Rot-induced formation of reactive oxygen species (ROS) in PC12 cells. (**B**–**D**) The combined effect of PCA and GB on the activities of GSH, SOD, and CAT in Rot-induced PC12 cells. Rot-induced PC12 cells were treated for 48 h with 25 μM GB or 0.6 mM PCA alone or in combination at the specified concentration. The data represent the means ± SDs of three independent experiments. ^#^ *p* < 0.05 and ^##^ *p* < 0.01 vs. the normal group, ** *p* < 0.01 and *** *p* < 0.001 vs. the GB-treated group. ^§§^ *p* < 0.01 vs. the PCA-treated group.

Reactive Oxygen Species (ROS) levels were monitored via changes in dichlorofluorescein (DCF). As shown in Figure 3A, ROS production significantly increased in Rot-induced PC12 cells, whereas synchronous treatment with drugs and Rot inhibited the production of ROS. The combined treatment with GB and PCA was better, reaching 54.13 ± 0.02%.

The overproduction of ROS will cause oxidative stress. GSH can stabilize sulfhydryl enzymes and protect haemoglobin or other cofactors from oxidative stress. Therefore, we evaluated the effect of each drug on intracellular GSH levels in Rot-induced PC12 cells. As shown in Figure 3B, the content of GSH reached 10.54 ± 1.36 μmol/g protein in the control group. After induction with Rot, GSH content in PC12 cells decreased to 3.26 ± 0.86 μmol/g protein. The addition of GB and PCA significantly increased the content of GSH in rotenone-damaged cells. Superoxide dismutase (SOD) and catalase (CAT) are crucial for the removal of reactive oxygen species. As shown in Figure 3C,D, the activities of both antioxidant enzymes decreased simultaneously after treating with Rot. The activities of SOD and CAT in treatment groups were significantly improved compared to the model group, indicating that the drugs effectively inhibited the decrease in intracellular antioxidant enzyme activity induced by Rot, and the effect of the combined treatment group was better than that of the single treatment group.

### 2.4. Combined Effects of GB and PCA on Expression of Mitochondrial Apoptosis Signaling Pathway Related Proteins in Rot-Induced PC12 Cells

**Figure 4:**
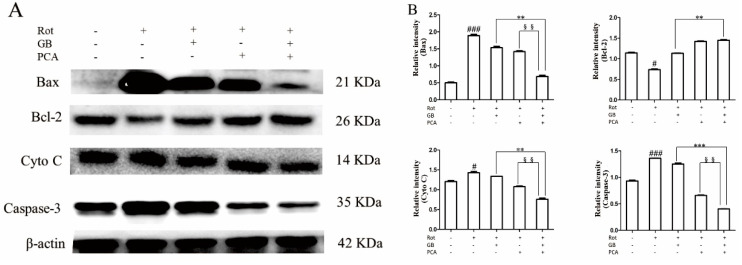
Combined effects of GB and PCA on the change in protein levels in Rot-induced PC12 cells. Rot-induced PC12 cells were treated for 48 h with 25 μM GB or 0.6 mM PCA alone or in combination at the specified concentration. (**A**) The pictures of western blot. (**B**) Intensity analysis of western blot. The data represent the means ± SDs. ^#^ *p* < 0.05 and ^###^ *p* < 0.001 vs. the normal group, ** *p* < 0.01 and *** *p* < 0.001 vs. the GB-treated group, ^§§^ *p* < 0.01 vs. the PCA-treated group.

Results of protein expression are shown in Figure 4A, and gray value analysis of protein expression is shown in Figure 4B. Compared with the control group, the expressions of Bax, Caspase-3, and Cytochrome C were significantly increased in the model group, while Bcl-2 expression was significantly decreased. The addition of drugs reversed the above results, and the effect was better after combined treatment with GB and PCA.

### 2.5. Combined Effects of GB and PCA on the Behavioural Outcome and Nerve Cell Injury of Parkinson Model Mice

The pole-climbing time and the suspension scores were measured for each group, and the results are shown in Table 1. The pole-climbing times of the normal group and the model group were 9.33 ± 0.54 s and 14.05 ± 1.52 s, respectively. The pole-climbing time of the drug-treated groups was significantly decreased compared with those of the model group. The pole-climbing time of the mice treated with the combination of GB and PCA decreased to 10.33 ± 1.20. The suspension scores of the normal group and the model group were 2.5 ± 0.04 and 1.95 ± 0.09, respectively. Compared with those of the model mice, the suspension scores of the mice treated with drugs were significantly improved. Moreover, the suspension of the mice treated with the combination of GB and PCA reached 2.25 ± 0.07, which was significantly different from that of the mice treated with GB or PCA alone.

**Figure 5:**
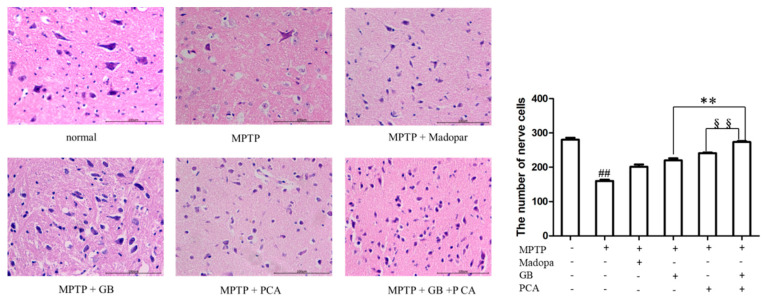
Morphological and quantitative changes in the neurons of mouse midbrains were observed by HE staining (200×). ^##^ *p* < 0.01 vs. the normal group, ** *p* < 0.01 vs. the GB-treated group, ^§§^ *p* < 0.01 vs. the PCA-treated group.

HE staining showed that in the normal group, the nuclei were clear, and the morphology of neurons in the substantia nigra of midbrain was complete. In the model group, the number of neurons was reduced, and the nucleoli were fuzzy. Compared with the model group, the number of neurons was increased in the drug-treated group, and the combined treatment group had the largest number of nerve cells. (See Figure 5).

### 2.6. Combined Effects of GB and PCA on Oxidative Stress Levels in the Midbrain of Parkinson’s Disease Mice

**Figure 6:**
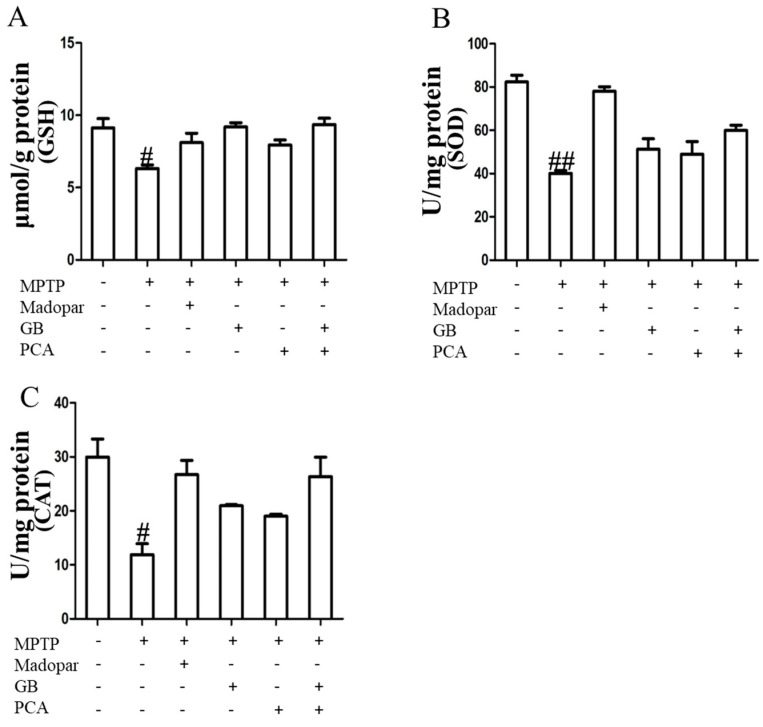
Combined effects of GB and PCA on the activity levels of GSH (**A**), SOD (**B**), and CAT (**C**) in the midbrain of Parkinson’s mice. Data were means ± SDs. ^#^ *p* < 0.05 and ^##^ *p* < 0.01 vs. normal group.

As shown in Figure 6, the activity levels of GSH, SOD, and CAT in the model group were significantly lower than in the normal group. After 21 days of treatment, the activity levels of them in the midbrain were significantly increased in the drug-treated groups compared with the model group. In addition, the combined treatment group had higher activity than the single-treatment group.

### 2.7. Combined Effects of GB and PCA on TH Expression in Substantia Nigra of Parkinson′s Disease Mice

**Figure 7:**
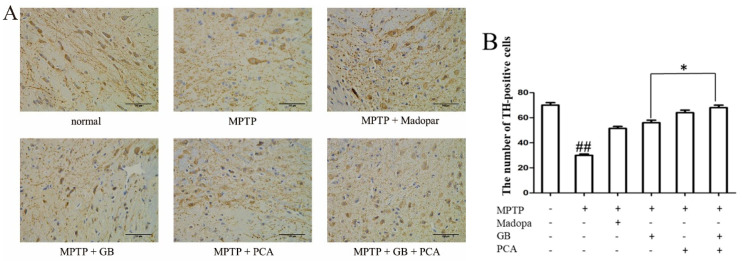
Combined effects of GB and PCA on TH expression in substantia nigra of Parkinson′s disease mice induced by MPTP (400×). (**A**) TH expression in substantia nigra. (**B**) Statistics of TH positive cells. ^##^ *p* < 0.01 vs. normal group. * *p* < 0.05 vs. GB-treated group.

Dopaminergic neurons in the substantia nigra of the mouse midbrain appeared as irregular triangles. As shown in Figure 7, compared with the normal group, the shape of TH-positive cells in the model group became blurred, the fibres became less and sparse, and the number of TH-positive cells was also decreased significantly (^##^ *p* < 0.01). TH-positive cells of the drug-treated groups had a darker cytoplasm and more fibres than those in the model group, and the number of positive cells was markedly increased. The number of positive cells in the combined treatment group was more than that in the single treatment group.

## 3. Discussion

At present, there have been many reports about the biological activity of Ginkgo Biloba Extract, and most of these reports focus on the main functional components (lactones and flavones). However, there are few reports on the synergistic effect of the main functional components and ginkgo organic acids.

Parkinson’s disease is the second-most common chronic degenerative disorder of the central nervous system. Environmental factors and genetics are important factors in the formation of Parkinson’s disease [17,18]. To date, the treatment of PD has not been perfected. There is no one treatment that can completely cure PD. More and more attention has been paid to traditional Chinese medicine for the treatment of PD. Both ginkgolide B and protocatechin have neuroprotective effects. Therefore, we used ginkgolide B (GB), one of the ginkgolides, and protocatechuic acid (PCA), one of the ginko organic acids, as the research objects to study the synergistic neuroprotective effect of GB and PCA in Parkinson’s disease.

In vitro, the MTT assay was used to test the impact of drugs on the cell viability of PC12 cells induced by rotenone. The results showed that GB and PCA protected PC12 cells from rotenone-induced injury. Moreover, the survival rate of PC12 cells upon the combination treatment with 25 μM GB and 0.6 mM PCA was higher than that upon treatment with GB or PCA alone. When the cytoplasmic membrane is damaged, intracellular LDH will be released into the culture medium, so detecting the LDH content can also be used to evaluate cell death [19]. In this study, it was found that the level of LDH in the cell culture medium of the model group was significantly increased, while the content of LDH in the treatment groups was significantly decreased. Moreover, the effect was better in the combined treatment group. The above experimental results were mutually verified and fully demonstrate that the combination treatment with GB and PCA can protect PC12 cells more effectively.

Rotenone, an inhibitor of mitochondrial complex I, can cause mitochondrial dysfunction. The mitochondrial electron transfer chain is not only associated with energy production, but also is associated with ROS production. Mitochondrial dysfunction can cause an overproduction of ROS [20]. Our research showed that when PC12 cells were treated with rotenone, the release of ROS increased. After treatment with GB and PCA, the above situation was reversed. The combined treatment group had the best inhibition effect. The overproduction of ROS will cause oxidative stress. Oxidative stress has been implicated as a major cause of cellular injures in neurodegenerative disorders [21]. Glutathione (GSH), an endogenous antioxidant peptide, plays an important role in the prevention of oxidative stress, thus protecting mitochondria and preventing apoptosis [22]. The study shows that the decrease of GSH in the midbrain of PD patients is positively correlated with the severity of PD [23,24,25]. Our results showed that GB and PCA augment GSH levels. Moreover, the overexpression of the components of the endogenous antioxidant system, such as SOD and CAT, may protect cells from damage induced by the simultaneous generation of NO and O_2•_^-^ [26]. Our results showed that the content of antioxidant enzymes enhanced significantly after treatment with GB or PCA alone or in combination, with the combined treatment being superior.

In addition to oxidative stress, apoptosis and mitochondrial dysfunction are also important factors leading to Parkinson’s disease [27]. Bax and Bcl-2, members of the Bcl family are involved in the positive and negative regulation of cell death [28]. Bcl-2 prevents cells from undergoing apoptosis induced by various stimuli in a wide variety of cell types [29], whereas others, such as Bax, promote and accelerate cell death. In addition, Bcl-2 is a kind of protein located on the outer membrane of mitochondria, which can stabilize the permeability of the membrane and maintain the integrity of mitochondria, thus inhibiting the release of cytochrome c and the activation of Caspase-3. In this study, we found that there was a significant increase in Bax expression as well as a decrease in bcl-2 expression after PC12 cells were treated with rotenone. Rotenone-mediated toxicity of PC12 cells also increased the expression of Cytochrome C and Caspase-3. These results were reversed by drug treatment. Moreover, combined treatment is more effective than single-treatment.

In vivo, the PD model was constructed by intraperitoneally injecting MPTP into C57BL/6 mice. During the experiment, it was found that the mice showed a series of behavioural changes to different degrees after MPTP was injected, including systemic static tremors, vertical hair, warped tails, unsteady gait, decreased activity, and other symptoms. The above behavioural changes lasted only for a few hours and gradually disappeared, possibly due to the strong compensatory ability of dopaminergic neurons in mice combined with the effects of acute modeling [30]. In this study, the behavioural changes in the Parkinson’s disease mice were quantitatively observed through the pole-climbing and suspension tests, which were used to detect the limb coordination of the mice. We found that there was a significant decline in the ability of the model group to climb and hang compared with that of the normal group. PD is also characterized by a decrease in dopaminergic neurons in the midbrain. We prepared pathological sections from the midbrain of Parkinson’s model mice. This effect was observed by HE staining under an inverted microscope. Compared with that in the normal group, the number of dopaminergic neurons in the MPTP group was significantly decreased, and the axons were shorter. The above behavioral and pathological results showed that the MPTP-induced PD model exhibited typical PD symptoms and pathological characteristics, thus making the model successful. After drug treatment, the above symptoms were relieved. Moreover, combined treatment with GB and PCA was more effective than single-treatment.

A large number of autopsy reports of Parkinson’s disease patients have shown that oxidative stress can significantly reduce the levels of antioxidant protection systems (GSH, SOD and CAT), reduce the activity of mitochondrial complex enzyme I, and eventually lead to the degeneration of dopaminergic neurons in the midbrain [31]. The results showed that the levels of GSH, SOD, and CAT in the midbrains of the mice in the model group were lower than those in the midbrains of the mice in the normal group, which was consistent with the relevant research results. The activity levels of SOD, CAT, and GSH in the midbrains of the mice in the combination treatment group were significantly improved.

Tyrosine hydroxylase (TH) is a rate-limiting enzyme in the synthesis of the neurotransmitter dopamine and catechol. Abnormal expression of TH is related to the occurrence of a variety of neurological diseases. Studies have found that a decrease in TH can be reflected by a decrease in the number of dopamine neurons [32]. Immunohistochemical analysis showed that TH expression in the PD model was significantly increased in treatment groups. Especially in the combination treatment group, the number of TH positive cells was the highest.

In summary, our results showed that the combination treatment with GB and PCA can inhibit the rotenone-induced cell death in PC12 cells by reducing oxidative stress levels and ameliorating mitochondrial dysfunction. It can also improve the motor ability of Parkinson mice, reduce MPTP-induced neuronal damage and the level of oxidative stress, and increase the expression of TH in Substantia Nigra. Moreover, the combined effect is better than the effect of individual treatment.

The results showed that the combination treatment of ginkgolide B and protocatechuic acid (the main component in organic acids) could enhance the therapeutic effect on Parkinson’s disease, which suggested that the organic acids could be selectively retained in the development of ginkgolide health products to enhance the therapeutic and health care effects of ginkgolide preparation. However, in this study, the specific pathways were not studied deeply enough. Therefore, further detailed studies are still needed before definite conclusions can be drawn.

## 4. Materials and Methods

### 4.1. Materials

Ginkgolide B (GB) and Protocatechuic acid (PCA) were purchased from Manster Biotechnology Co., Chengdu, China. The purity of ginkgolide B and protocatechuic acid were more than 99% (HPLC). Rotenone (Rot) and 1-methyl-4-phenyl-1, 2, 3, 6-tetrahydropyridine (MPTP) were purchased from Aladdin Biochemical Technology Co., Shanghai, China. Madopar is a drug used to treat Parkinson’s disease. It has been used as a positive drug in many animal models of Parkinson’s disease.

### 4.2. Cell Culture and Assay of Cell Viability

PC12 cells were purchased from the Typical Culture Preservation Committee Cell Bank, of the Chinese Academy of Sciences. The cells were maintained in Dulbecco’s modified Eagle′s medium (DMEM) with 10% heat-inactivated fetal bovine serum (from Gibco, Grand Island, NY, USA), penicillinand streptomycin (from Beyotime, Shanghai, China). The cells were grown at 37 °C in an atmosphere containing 5% CO_2_.

Cell viability was measured via 3-(4, 5-dimethylthiazol-2-yl)-2, 5-diphenyltetrazolium bromide (MTT) assay. PC12 cells were seeded in 96-well plates with a density of 5 × 10^4^ cells/mL. When the cells reached 70% fusion, cells were incubated with GB (6.25, 12.5, 25, 50, 100 μM) or PCA (0.15, 0.3, 0.6, 1.2 mM) or in combination in DMEM for half an hour, and then Rotenone (Rot) was added to co-culture for 48 h. Then, 4 mg/mL MTT was added to the cells and removed after 4 h. Also, 200 μL dimethyl sulfoxide (DMSO, Aladdin, Shanghai, China) was used to lyse the sediment. The 96-well plates were stirred for 10 min on a microtiter plate shaker, and the absorbance was read at 540 nm.

### 4.3. Morphological Analysis of PC12 Cells

The cells were seeded in 6-well plates with a density of 2 × 10^5^ cells/mL. When the cells had adhered to the walls and were in good condition, the drugs and rotenone were added. The cells were cultured for another 48 h and then photographed with an inverted microscope.

### 4.4. LDH Release

PC12 cells were seeded in 96-well plates with a density of 5 × 10^4^ cells/mL. PC12 cells were digested with trypsin and collected by centrifugation at 1000× *g* for 5 min. The homogenate was centrifuged at 3500× *g* for 10 min. Then, the supernatant was used to test the activity according to the manufacturer’s instructions.

### 4.5. Detection of Intracellular Reactive Oxygen Species (ROS)

The cells were seeded in 6-well plates with a density of 2 × 10^5^ cells/mL. To analyse intracellular ROS levels, the PC12 cells were incubated with a fluorescent DCFH-DA probe for 20 min at 37 °C at a final concentration of 10 μM in the dark. The fluorescence intensity was tested at an emission wavelength of 525 nm and an excitation wavelength of 488 nm by an automated microplate spectrophotometer (SpectraMax 190, San Francisco, CA, USA).

### 4.6. Determination of Antioxidant Enzyme Activity

PC12 cells (2 × 10^5^ cells/mL) were seeded in 6-well plates. The content of GSH was measured on the basis of the manufacturer’s instructions (Jiancheng, Biotechnology, Nanjing, China). Ultimately, the absorbance was evaluated at 405 nm. Superoxide dismutase (SOD) and Catalase (CAT) activity were measured according to the test kit (Jiancheng, Biotechnology, Nanjing, China). The principle of detecting SOD activity is that the reaction between xanthine and xanthine oxidase produces O_2•_^-^, forming nitrite, which turns purplish-red under the action of a colour reagent. The OD value was measured via spectrophotometer. CAT can directly decompose its substrate hydrogen peroxide (H_2_O_2_) under certain conditions, such that as the concentration of H_2_O_2_ in the reaction solution gradually decreases, the corresponding OD value also gradually decreases.

### 4.7. Protein Extraction and Western Blotting

PC12 cells were seeded in 6-well plates at a density of 2 × 10^5^ cells per well. The cells were lysed by using WB and IP lysis buffer supplemented with a protease inhibitor cocktail to release whole-cell proteins. A total of 40 μg of total protein was taken from each group and separated by 15% SDS-PAGE electrophoresis. The proteins were transferred to a PVDF membrane after electrophoresis. The membranes were blocked with milk powder for 1 h at room temperature. Then the membrane was blocked and probed with antibodies against Bax (1:500, GB11690, servicebio), Bcl-2 (1:500, 26593-1-AP, proteintech), Cyto C (1:500, GB12080, servicebio), Caspase-3 (1:1000, 9662, CST), β-actin (1:1000, GB12001, servicebio) at 4 °C overnight. After rinses in TBST, the corresponding secondary antibody was added, and the samples were incubated for 2 h at room temperature. After rinses in TBST, ECL solution was added for development. Images were collected with the LAS-3000 chemiluminescence imaging system.

### 4.8. Animals and Drug Treatment

Considering the high sensitivity of black male mice to MPTP, we purchased eight-week-old adult male C57BL/6 mice (weighing 18–22 g) from the Experimental Animal Center of Qinglongshan Experiments Animal Factory (Nanjing, China). The animals were group-housed in rearing cages with water and food under a 12-h light/dark cycle. All experiments and animal care protocols were conducted in accordance with the National Institutes of Health guide for the care and use of laboratory animals.

Mice were divided into the following six groups of 12 mice each based on weight after one week of acclimation: (1) the normal group; (2) the MPTP (30 mg/kg) group; (3) the MPTP + Madopar (75 mg/kg) group; (4) the MPTP + GB (ginkgolide B, 20 mg/kg) group; (5) the MPTP + PCA (protocatechuic acid, 5 mg/kg) group; and (6) the MPTP + GB + PCA (ginkgolide B, 20 mg/kg; protocatechuic acid, 5 mg/kg) group. Each group was given the appropriate drugs by gavage in a volume of 0.1 mL/10 g body weight once per day for 21 consecutive days. The model group and the treatment group were intraperitoneally administered MPTP (30 mg/kg) once per day for 7 continuous days from the 8th day. The normal group was injected with the same amount of PBS.

### 4.9. Behavioural Test

Within one day after the completion of modelling, the motor dysfunctions of each group were tested via the pole-climbing and suspension tests. Before the formal test, a 2-day training protocol was conducted to adapt animals to the experimental environment.

The pole-climbing test was carried out according to the literature [33]. In short, a wooden pole 1-cm in diameter and 50-cm in length was placed on a foam board and wrapped with gauze to prevent slipping. The mice were put on the top of the pole and allowed to climb down free from disturbance three times within a 5-min interval. We recorded the time it took for each mouse to climb down the pole. If the mouse stopped halfway or turned around to climb to the top of the pole, the experiment was invalid and carried out again.

For the suspension test, the mice were suspended from a horizontal wire and were awarded 3 points for grasping the wire with two paws and 2 points for grasping the wire with one paw. If a mouse could not hold the wire with its paws, 1 point was awarded.

### 4.10. Tissue Preparation

The mice were sacrificed under diethyl ether anaesthesia after the behavioural tests. Whole brains were quickly removed and stored at −80 °C for detection of the antioxidant index. Four brain samples from each group were collected following perfusion through the heart and fixed overnight in 4% paraformaldehyde at room temperature. The substantia nigra of these samples were prepared for HE staining and immunohistochemistry [34].

### 4.11. Statistical Analysis

Data processing used the GraphPad Prism 5 software. The data were expressed as mean ± SD. One-way anova was used between the groups, and *p* < 0.05 was considered as statistically significant. In this paper, the symbol (#) represents the significant comparison between the model group and the control group. The symbol (*) and the symbol (§) represent the significance comparison of combined treatment group with GB-treated and PCA-treated groups, respectively. The combined effects of the two drugs were analysed by Q value [35]. Q = E_a+b_/(E_a_ + E_b_ − E_a_ × E_b_), where E_a_ and E_b_ represent the inhibition rate when the two drugs act alone, and E_a+b_ represents the inhibition rate when they act together. The Q value is the ratio of the two; Q > 1.15 means the ratio is synergistic.

## Figures and Tables

**Table 1:** Behavioural Testing.

Group	Climbing Time/s	Score of Suspension Test/Score
Normal	9.33 ± 0.54	2.5 ± 0.04
MPTP	14.05 ± 1.52 ^###^	1.95 ± 0.09 ^###^
MPTP + Madopar	9.95 ± 0.74	2.28 ± 0.22
MPTP + GB	10.69 ± 1.59	2.19 ± 0.04
MPTP + PCA	11.17 ± 1.88	2.11 ± 0.04
MPTP + GB + PCA	10.33 ± 1.20	2.25 ± 0.07 *^,§§§^

Note: ^###^ *p* < 0.001 vs. the normal group, * *p* < 0.05 vs. the GB-treated group, ^§§§^ *p* < 0.001 vs. the PCA-treated group.

## Abbreviations

The following abbreviations are used in this manuscript:

EGB	Ginkgo biloba extract
GB	Ginkgolide B
PCA	Protocatechuic acid
Rot	Rotrnone
MPTP	1-methyl-4-phenyl-1, 2, 3, 6-tetrahydropyridine
PD	Parkinson’s disease
LDH	lactate dehydrogenase
ROS	Reactive Oxygen Species
SOD	Superoxide dismutase
CAT	Catalase
TH	Tyrosine hydroxylase
